# Phenotypic variability and population structure analysis of Tanzanian free-range local chickens

**DOI:** 10.1186/s12917-020-02541-x

**Published:** 2020-09-29

**Authors:** James R. Mushi, Gaspar H. Chiwanga, Esinam N. Amuzu-Aweh, Muhammed Walugembe, Robert A. Max, Susan J. Lamont, Terra R. Kelly, Esther L. Mollel, Peter L. Msoffe, Jack Dekkers, Rodrigo Gallardo, Huaijun Zhou, Amandus P. Muhairwa

**Affiliations:** 1 Department of Veterinary Medicine and Public Health, Sokoine University of Agriculture, Morogoro, Tanzania; 2 Department of Animal Science, University of Ghana, Accra, Ghana; 3 Department of Animal Science, Iowa State University, Ames, IA 50011 USA; 4 School of Veterinary Medicine, University of California, Davis, 95616 USA; 5 Department of Animal Science, University of California, Davis, 95616 USA

**Keywords:** Free-range local chickens, Phenotypic diversity, Genetic diversity, Population structure

## Abstract

**Background:**

Free-range local chickens (FRLC) farming is an important activity in Tanzania, however, they have not been well-characterized. This study aimed to phenotypically characterize three Tanzanian FRLCs and to determine their population structure. A total of 389 mature breeder chickens (324 females and 65 males) from three popular Tanzanian FRLC ecotypes (Kuchi, Morogoro-medium and Ching’wekwe) were used for the phenotypic characterization. Progenies of these chickens were utilized to assess population structure. The ecotypes were collected from four geographical zones across Tanzania: Lake, Central, Northern and Coastal zones. Body weights and linear measurements were obtained from the mature breeders, including body, neck, shanks, wingspan, chest girth, and shank girth. Descriptive statistics were utilized to characterize the chickens. Correlations between the linear measurements and differences among the means of measured linear traits between ecotypes and between sexes were assessed. A total of 1399 progeny chicks were genotyped using a chicken 600 K high density single nucleotide polymorphism (SNP) panel for determination of population structure.

**Results:**

The means for most traits were significantly higher in Kuchi relative to Ching’wekwe and Morogoro-medium. However, shank length and shank girth were similar between Kuchi and Morogoro-medium females. All traits were correlated with the exception of shank girth in Morogoro-medium. Admixture analyses revealed that Morogoro-medium and Ching’wekwe clustered together as one population, separate from Kuchi.

**Conclusions:**

Phenotypic traits could be used to characterize FRLCs, however, there were variations in traits among individuals within ecotypes; therefore, complementary genomic methods should be considered to improve the characterization for selective breeding.

## Background

Poultry plays an important role in the livelihoods [1] of communities in Africa. It is among the most prevalent livestock produced in Tanzania, and chickens account for approximately 94% of poultry raised by farmers [2]. Free-range local chickens (FRLCs) have been produced in Tanzania for many years [3]. In rural communities, the production is mainly for subsistence [2, 4]. Commercial poultry production is common in urban areas where farmers typically raise exotic breeds in intensive systems.

The FLRCs are relatively adapted to and resilient to stressful conditions, including harsh weather and disease [5–8]. They can be produced with minimal resources, such as shelter, feed, and veterinary services. As a result, they serve as important sources of animal protein and household income, especially for resource-poor marginalized rural communities. The FRLCs and their products are also socially and culturally accepted across different Tanzanian communities. Despite their importance, research on improving productivity of the FRLCs is lacking [9]. Tanzania has over 17 ecotypes of indigenous chickens [10, 11]. Most of these ecotypes have not been well-characterized and their production potential is poorly understood.

There are several methods used to characterize animals ranging from linear measurement of morphological traits to the use of molecular techniques [12]. For instance, morphological measurements have been used to characterize and compare various poultry breeds [13, 14] and microsatellites have been used to determine the origin of African chickens [15–17]. In addition, single nucleotide polymorphism (SNP) makers have been used to compare the best method for ascertaining diversity among chickens [18], to cluster the genomes of commercial and non-commercial chicken breeds [19] and to investigate the genetic structure of chicken populations [20]. Climatic conditions and other stressors are highly variable across Tanzania. As a result, FRLC populations may develop different adaptation mechanisms leading to spatial differences in population structure. The extensive management system used to rear FRLCs also allows for random mating leading to panmictic populations [21] with no clearly defined chicken types, strains or lines. The aim of this study was to characterize three Tanzanian FRLC ecotypes using linear body measurements and population structure analysis. Information generated through this study will inform on selection programs to improve FRLC production in Tanzania.

## Results

### Phenotype characterization

The results of the effects of chicken ecotype and sex on the morphometric and body weight measurement traits are presented in Table 1. Both ecotype and sex effects had a significant influence on traits with the exception of shank girth (SG). Interactions between sex and ecotype effects were only significant for the CG, WS and SL. Males were also significantly different from females for the BL, NL, CG, WS and BW measurements. Similarly, there were significant differences between ecotypes for the BL, NL, CG, WS, SL and BW. Least square means (LSmeans) of the body measurements along with their standard errors (±SE) are shown in Table 2. The Kuchi ecotype had higher mean values for BL, NL, and BW measurements compared to Ching’wekwe and Morogoro-medium ecotypes, and measurements for these traits were significantly higher in male chickens. The LSmeans for the CG, WS and SL were significantly higher in males across all ecotypes. For the Morogoro-medium chickens, the LSmean for the SL was higher in males, but the difference was not statistically significant. Significant differences in LSmeans for the BL, NL and BW were detected across the ecotypes with the highest LSmeans in Kuchi, followed by Morogoro-medium and Ching’wekwe. There were no significant differences in the SG between sexes across the ecotypes. Overall, males had higher mean measurements across the ecotypes. However, there was individual variation within ecotypes for both sexes that were beyond the means of the ecotypes, where measurements overlapped with other ecotypes. These results indicate that Kuchi chickens were heavier and longer/taller than Morogoro-medium and Ching’wekwe chickens were the shortest and lightest. Linear measurements and body weights within each ecotype were positively correlated except for Morogoro-medium where the SG showed no significant correlation with any other traits (Tables 3, 4 and 5).

**Table 1:** Analysis of variance *p*-values for measured traits as affected by the ecotype and sex

Effects	BL	NL	CG	WS	SL	SG	BW
Ecotype	<2e-16^***^	<2e-16^***^	<2e-16^***^	<2e-16^***^	<2e-16^***^	0.1266	<2e-16^***^
Sex	<2e-16^***^	<2e-16^***^	<2e-16^***^	<2e-16^***^	<2e-16^***^	0.0577.	<2e-16^***^
Ecotype: sex	0.526	0.526	0.0106^**^	0.0194^*^	0.000000678^***^	0.7096	0.426

*BL* Body length, *NL* Neck length, *CG* Chest girth, *WS* Wingspan, *SL* Shank length, *SG* Shank girth, *BW* Body weight, ***p < 0.001, **p ≤ 0.01, *p ≤ 0.05

**Table 2:** Least square means (LSmeans±SE) with standard error of measured traits among the FRLC

Trait	Sex		Ecotype	
		Kuchi	Ching’wekwe	Morogoro- medium
BL	M	50.9 ± 0.62^a^	46.1 ± 0.95^b^	48.30 ± 0.41^c, f^
	F	45.2 ± 0.48^a, d^	43.7 ± 0.22^b, e^	46.80 ± 0.72^c^
NL	M	19.4 ± 0.54^a^	17.0 ± 0.54^b^	17.40 ± 0.28^c, f^
	F	18.0 ± 0.29^a, d^	15.8 ± 0.17^b, e^	16.70 ± 0.45^c^
CG	M	35.30 ± 0.59^a^	31.1 ± 0.45^b^	34.0 ± 0.25^c, f^
	F	29.30 ± 0.20^a, d^	29.0 ± 0.17^b, e^	31.86 ± 0.44^c^
WS	M	47.7 ± 0.78^a^	43.1 ± 0.75^b^	42.74 ± 0.55^c^
	F	45.7 ± 0.82^a, d^	40.0 ± 0.47^b, e^	42.60 ± 0.77^c, f^
SL	M	11.4 ± 0.28^a^	10.2 ± 0.25^b^	10.30 ± 0.16^c^
	F	10.5 ± 0.16^a, d^	9.0 ± 0.09^b, e^	9.90 ± 0.19^c^
SG	M	5.1 ± 0.17^a^	4.1 ± 0.12^a^	4.6 ± 0.06^a^
	F	4.7 ± 0.06^a^	3.9 ± 0.04^a^	4.4 ± 0.13^a^
BW	M	2152.4 ± 50.25^a, d^	1687.6 ± 84.02^b^	2090.4 ± 38.55^c, f^
	F	1575.47 ± 91.37^a^	1162.5 ± 30.65^b, e^	1455.7 ± 68.23^c^

Same superscript small letters indicate no significant difference between mean measurements. First superscript small letters compare among ecotype where the second superscript small letter compares between sex. *M* males and *F* females, *BL* Body length, *NL* Neck length, *CG* Chest girth, *WS* Wingspan, *SL* Shank length, *SG* Shank girth, *BW* Body weight

**Table 3:** Correlations among measured traits in Kuchi ecotype at *p* ≤ 0.05

Measured trait	BL	NL	CG	WS	SL	SG	BW
BL	1						
NL	0.8***	1					
CG	0.75***	0.68***	1				
WS	0.77***	0.75***	0.77***	1			
SL	0.83***	0.77***	0.78***	0.85***	1		
SG	0.78***	0.68***	0.87***	0.75***	0.77***	1	
BW	0.76***	0.62***	0.808***	0.63***	0.67***	0.83***	1

*BL* Body length, *NL* Neck length, *CG* Chest girth, *WS* Wingspan, *SL* Shank length, *SG* Shank girth, *BW* Body weight, ***p < 0.001

**Table 4:** Correlations among measured traits in Ching’wekwe ecotype at *p* ≤ 0.05

Measured trait	BL	NL	CG	WS	SL	SG	BW
BL	1						
NL	0.65***	1					
CG	0.62***	0.41***	1				
WS	0.43***	0.39***	0.34***	1			
SL	0.57***	0.45***	0.34***	0.47***	1		
SG	0.71***	0.53***	0.64***	0.36***	0.36***	1	
BW	0.69***	0.42***	0.77***	0.38***	0.45***	0.8***	1

*BL* Body length, *NL* Neck length, *CG* Chest girth, *WS* Wingspan, *SL* Shank length, *SG* Shank girth, *BW* Body weight, ***p < 0.001

**Table 5:** Correlations among measured traits in Morogoro-medium ecotype at *p* ≤ 0.05

Measured trait	BL	NL	CG	WS	SL	SG	BW
BL	1						
NL	0.57***	1					
CG	0.75***	0.46***	1				
WS	0.74***	0.55***	0.69***	1			
SL	0.72***	0.59***	0.66***	0.85***	1		
SG	0.09	0.04	0.07	0.1	0.07	1	
BW	0.74***	0.41***	0.91***	0.66***	0.62***	0.11	1

*BL* Body length, *NL* Neck length, *CG* Chest girth, *WS* Wingspan, *SL* Shank length, *SG* Shank girth, *BW* Body weight, ***p < 0.001

### Population structure evaluation

**Fig. 1:**
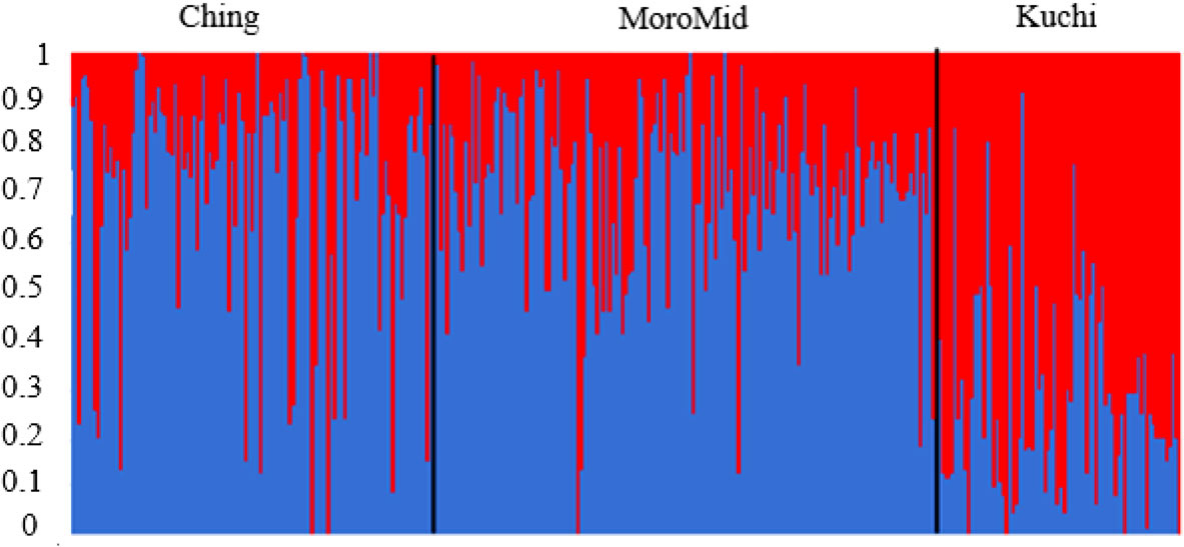
Admixture analysis plot showing mixed ancestry among individuals for the three chicken ecotypes; Ching = Ching’wekwe, MoroMid = Morogoro-medium, Kuchi = Kuchi (Source- Walugembe et al., 2019)

**Fig. 2:**
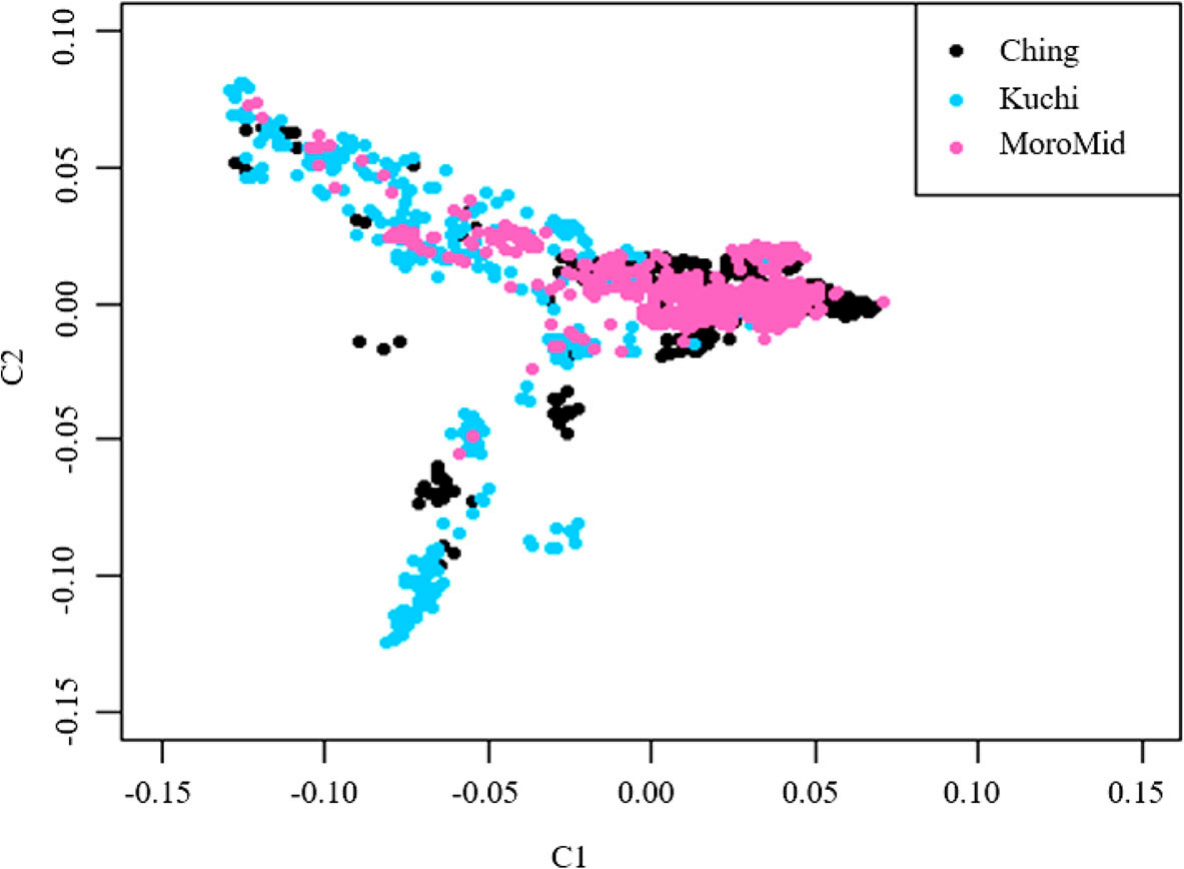
Multi-dimensional scaling (MDS) plot showing the distribution of chickens in three clusters of the sampled population. Ching = Ching’wekwe, MoroMid = Morogoro-medium, Kuchi = Kuchi (Source- Walugembe et al., 2019)

The admixture analysis for the genetic population structure of the selected Tanzanian FRLC using SNP genotypes indicated evidence of admixture among the FRLC ecotypes (Fig. 1). From the analysis, the three chickens’ ecotypes clustered into two populations instead of distinct three ecotypes. The Ching’wekwe and Morogoro-medium ecotypes had higher average population proportions of population two (0.78 and 0.75 for Ching’wekwe and Morogoro-medium, respectively), compared to Kuchi that had a higher average proportion (0.67) of population one as shown in Table 6. Admixture population structure results were supported by the multi-dimensional scaling (MDS) plot (Fig. 2), with Ching’wekwe and Morogoro-medium clustering more closely together compared to Kuchi.

**Table 6:** Average proportions of admixture per ecotype

Chicken ecotype	Proportions (K = 2)	
	Population1	Population2
Ching’wekwe	0.78	0.22
Kuchi	0.33	0.67
Morogoro-medium	0.75	0.25

## Discussion

This study was designed to evaluate linear measurements which could be used to phenotypically characterize three selected Tanzanian FRLC ecotypes based on their morphometrics, key criteria that farmers in Tanzania use to select chickens for breeding purposes. Further, it aimed to enhance understanding of the population structure of the selected FRLC ecotypes using single nucleotide polymorphism (SNP) genetic markers. These results provide a deeper insight into population structure of the FRLCs to complement the use of phenotypic selection. Proper characterization of the FRLC ecotypes, which has not been previously performed due to a lack of known parent stock and reliable source of day-old chicks [22], will promote their commercialization while improving their productivity through aiding in genetic selection of higher performing chickens. In this study, measurements of the BL, NL, CG, SL, SG, WS and BW were evaluated and compared among Kuchi, Ching’wekwe and Morogoro-medium Tanzanian FRLC ecotypes. Ecotype and sex had a significant influence on most of the physical measurements of the chickens (Table 1), similar to observations by Alabi et al. [12] for indigenous chickens in South Africa. The males in all groups had the highest mean scores for all the measurements compared to the females (Table 2).

In this study, the Kuchi ecotype had relatively higher mean values for most of the measured traits as compared to the Ching’wekwe and Morogoro-medium ecotypes for both sexes. Ching’wekwe had the least mean values for all measurements. The findings in this study are similar to findings by Lweramila et al. [4] who compared the performance of Kuchi and Morogoro-medium under pure extensive management systems as well as findings by Magonka et al. [23] which revealed that Kuchi had the highest scores for most measurements compared to Horasi, Naked-neck and Frizzled ecotypes. Based on the results and the physical appearance of the chickens, the Ching’wekwe were shorter than Morogoro-medium and Kuchi, probably owing to their proportionately shorter shanks and body parts. Kuchi on the other hand were observed to have a higher upright stature than the other two ecotypes. Apart from the observed mean variations between ecotypes, there were also large variations observed within ecotypes such that there are some individuals within each group that fell into extremes beyond the group means, thus, overlapping with individuals in other groups. The extreme measurements seen with some individual birds might be a result of random mating in the extensive free-ranging system that leads to admixtures of genotypes and that might have produced the intermediary traits observed in these individuals.

Body weights at maturity were also measured among the chicken types to complement the characterization. The results of this study corroborate previous findings by Lweramila at al [4] and Lyimo et al. [17] working with chickens in an extensive husbandry system where Kuchi weighed more than the other FRLC types. As expected, the males had higher mean body weights than the females as observed in many feeding trials [24]. However, some females in the current study had higher weights than males, probably due to changes in body physiology during laying periods whereby there is increase in the uterus size, fat deposition and increased feed intake [25]. Correlation analyses among the measured traits within ecotypes were positive and high for all traits in Kuchi and Morogoro-medium similar to observations by Alabi et al. [12]. However, there was no significant correlation between the SG and other measured body traits (Table 5). Results of the measurable phenotypic features used in this study could place the chickens into three suggestive ecotypes as they are known from their places of origin suggesting that phenotypic measurable features observed in mature FRLC may be used to complement other methods for identification of chicken ecotypes, especially among the three ecotypes used in this study.

**Fig. 3:**
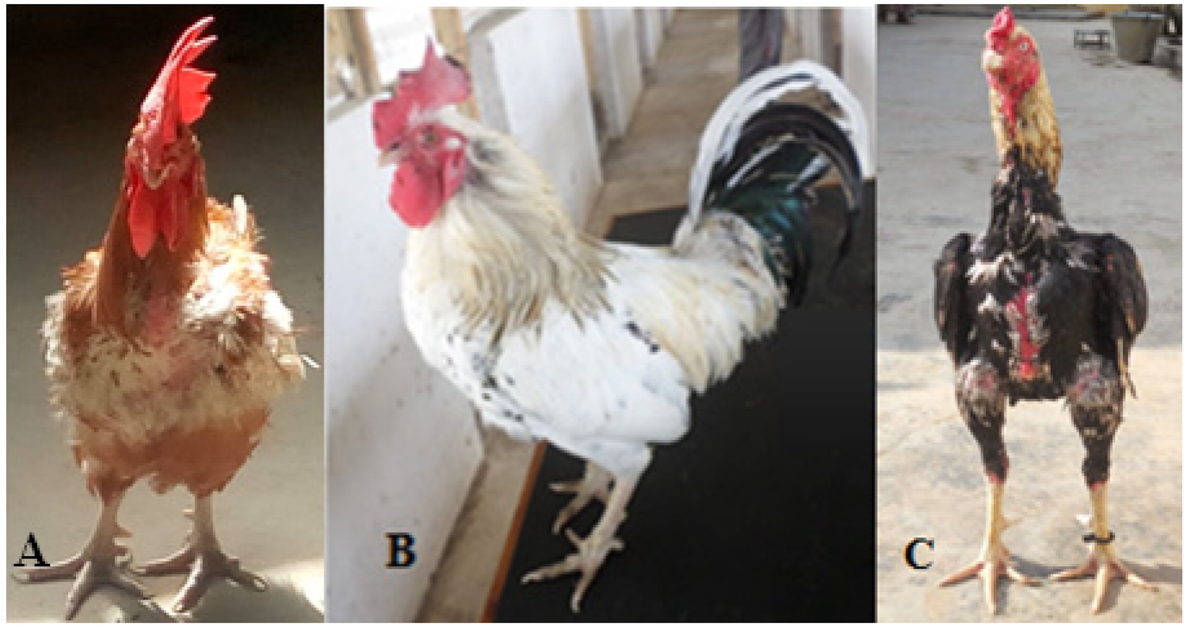
**a**, **b** and **c** are Ching’wekwe, Morogoro-medium and Kuchi chicken ecotypes respectively

The population structure analysis using admixture analysis placed the three selected FRLC ecotypes into strata of two populations instead of three ecotypes as initially perceived from the phenotypic study. A similar study using microsatellite genetic markers from five Tanzania local chicken ecotypes (Unguja, Pemba, Ching’wekwe, Morogoro-medium and Kuchi), [17] revealed similar findings in which Ching’wekwe and Morogoro-medium clustered together as one population while Kuchi stood as a separate population. Ching’wekwe and Morogoro-medium are found in areas with similar climatic conditions with no natural separation between the chicken types (Fig. 3). The lack of geographic barriers, the purchase of seeder flocks from region to region and the free-range management system of the FRLC in Tanzania might have increased the chances of interbreeding between these two ecotypes resulting in one population rather than two. The Kuchi are more adapted to regions of the Lake and Central zones which are cooler and more humid regions than the Morogoro and Tanga regions where the Ching’wekwe and Morogoro-medium ecotypes are adapted. Studies by Oka et al. [26] revealed that the Shamo chicken types of the Shikoku islands and Kuchi share the same mitochondrial DNA haplotypes. Also, Komiyama et al. [27] reported that the conformation of Kuchi beak is hooked or parrot-like and sharp like the Shamo chickens of Japan. These studies suggest that the Kuchi chickens might have originated from Japan and formed a breeding colony in the Lake and Central zones where they are adapted. Further, research by Lyimo et al. [17] into the origins of Tanzanian local chickens using microsatellite markers found that the Kuchi are the least genetically diverse chicken type among five Tanzanian chickens investigated in the study.

## Conclusions

With an exception of the SG, the mean linear measurements of traits used in the current study were significantly different between ecotypes. This indicates that phenotypic trait can be used to identify the different chicken ecotypes. The strong correlations among the linear measurements show that selection for one trait means a selection for the other traits, with the exception of SG in the Morogoro-medium ecotype, which was poorly correlated with other traits.

Individual variation in the measurements within ecotypes with overlap of extreme values between ecotypes was observed, making it difficult to predict a chicken’s ecotype. As a result, additional information such as history should be used to complement the phenotypic characterization. However, from the results, it is difficult to use phenotypic measurable features to assign the FRLC to a particular genetic chicken population. Thus, the selection of FRLC for breeding purposes would be more canonical with use of genomic tools compared to the customary phenotypic methodologies in use by the FRLC farmers in the country.

## Methods

Procedures for handling the experimental animals were approved by the Institutional Animal Care and Use Committee (IACUC) of the University of California Davis (# 20831).

### Study area

**Fig. 4:**
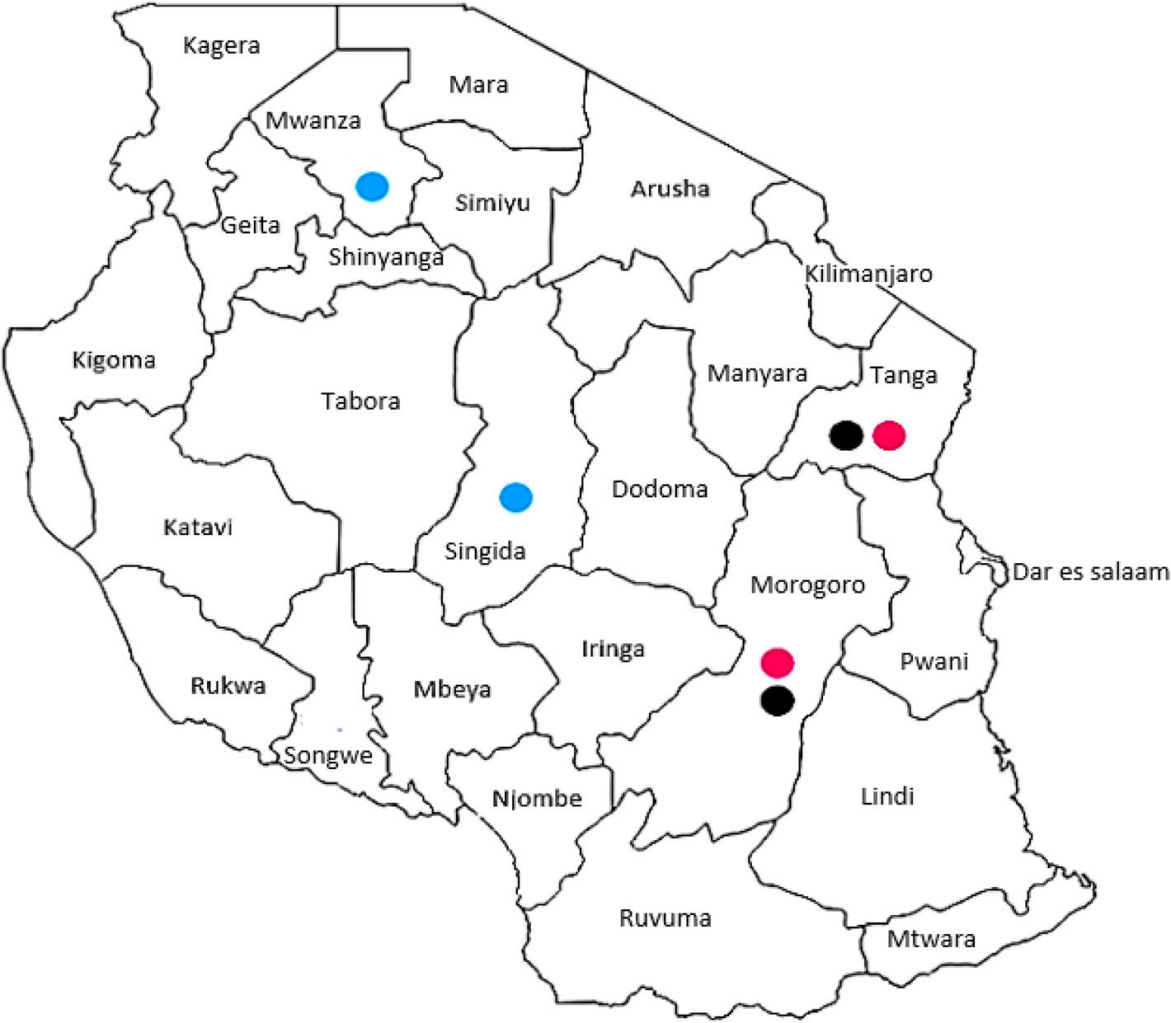
Geographical origins of Kuchi (blue), Morogoro-medium (purple) and Ching’wekwe (Black) chickens in Tanzania (https://d-maps.com/carte.php?num_car=36219&lang=en, 2/3/2020)

Experiments were conducted at Sokoine University of Agriculture (SUA) in Morogoro, Tanzania using facilities of the Department of Animal, Aquaculture and Range Sciences (DAARS). Three Tanzanian FRLC ecotypes (Kuchi, Ching’wekwe and Morogoro-medium) (Fig. 3) were randomly sampled from different zones with varying climatic conditions across the Tanzania mainland. The locations and weather conditions of the different regions and zones are shown in Table 7. Ching’wekwe and Morogoro-medium were sampled from regions in close proximity (Morogoro and Tanga regions) to the Coastal and Northern zones; whilst Kuchi were sampled from the Lake and Central zones (Mwanza and Singida regions; Fig. 4) [28].

**Table 7:** Regional sources of parent stock FRLC

FRLC	Regions	Location (DD)	Altitude (m)	Av. Temp (°C)^a^	Av. Humidity (%)^b^
Ching’wekwe	Morogoro	−5.5°, 34.5°	213	24.6	75%
	Tanga	−5.0667°, 39.1°	22	28.0	76%
Kuchi	Mwanza	−2.85°, 33.083°	1363	23.3	76%
	Singida	−5.483°, 34.483°	1508	22.0	74%
Morogoro-medium	Morogoro	−5.5°, 34.5°	213	24.6	75%
	Tanga	−5.0667°, 39.1°	22	28.0	76%

^a^Average temperature per year, ^b^Average humidity per year

### Experimental chickens

A flock of 389 FRLCs (324 females and 65 males) of the three ecotypes were randomly collected from village households in four regions of Tanzania (Tanga, Morogoro, Singida, and Mwanza regions) and were used to establish a breeding parent stock. Identification of chicken ecotypes was performed as previously described by Msoffe et al., and Guni and Katule [10, 11]. Each chicken was marked with a numbered aluminium wing tag. For each chicken ecotype, a male was placed separately in a pen with 6 to 10 females in a deep litter floor pen. The parent flocks were fed on maize-based layer diets with ad libitum access to water. Routine vaccinations against endemic diseases (Newcastle disease and infectious bursal disease) were administered [29, 30]. Worm infestations, ectoparasites and coccidiosis were treated/controlled using anthelmintics (piperazine DiHCl®, Kepro, Holland), pesticides (imidacloprid Confidor®, Bayer, Holland), and coccidiostats (Trisulmycine®, Laprovet, France), respectively.

### Progeny generation chickens

Experimental chicken progenies were established using eggs collected from the parent stock for up to 10 consecutive days. The eggs were labelled with numbers corresponding to a sire and temporarily stored at 18 °C before incubation at 60% humidity and 37 °C. On day 18 post-incubation, the eggs were transferred to a hatcher with special racks with cubical separations corresponding to sire identity to avoid mixing of chick progenies at hatching. Day old chicks were wing-tagged, weighed and transferred to a bio-secure deep litter floored experimental chicken house where they were fed on commercial chick mash and ad-libitum water access. Treatment for coccidiosis was performed as needed to control outbreaks in the flock. A total of 1399 chicks (477 Ching’wekwe, 315 Kuchi, and 607 Morogoro-medium) were produced following five rounds of incubation and hatching for use in the population structure analysis of the three FRLC ecotypes.

### Phenotypic linear measurements

**Fig. 5:**
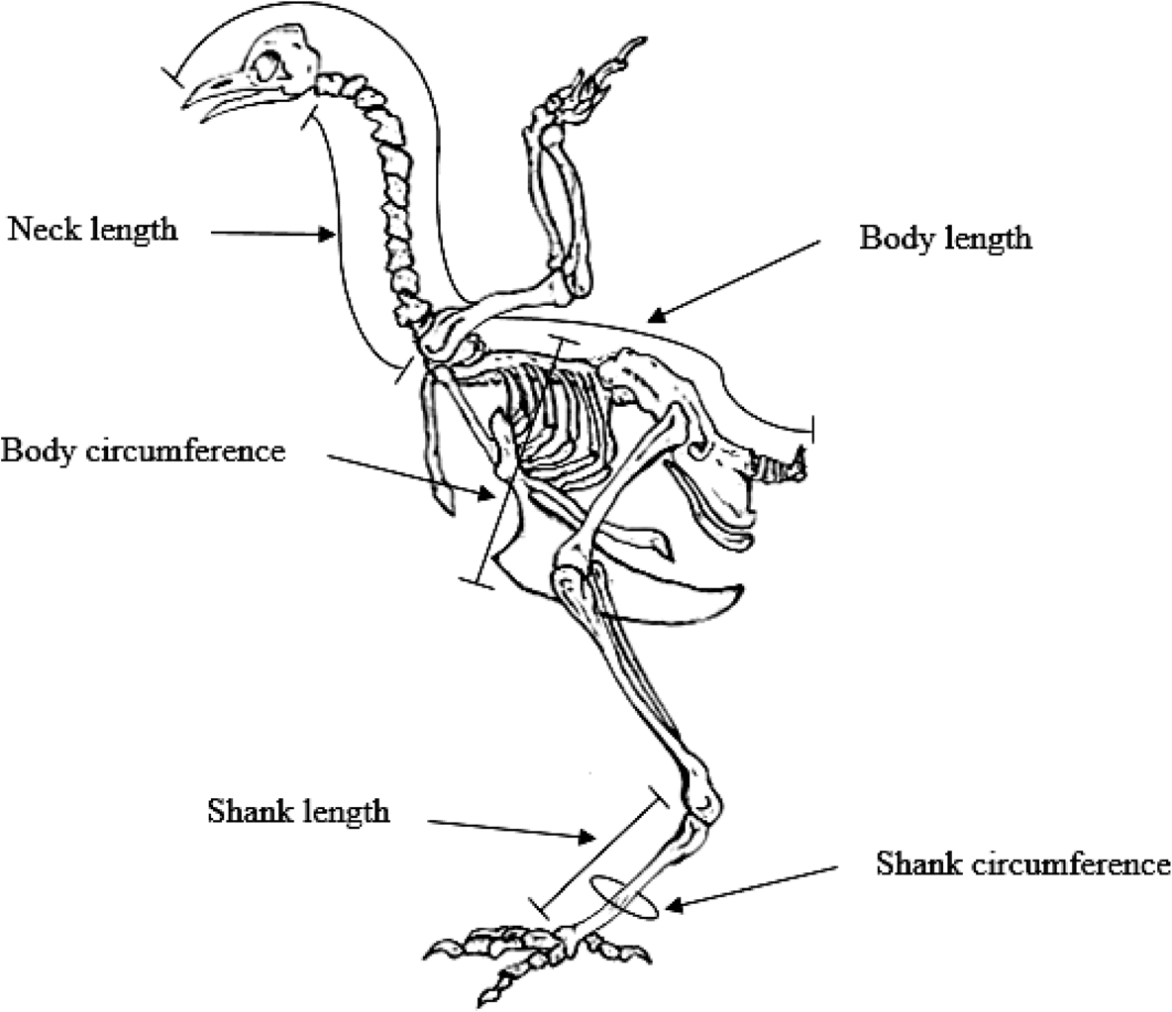
Pictorial representation of where various linear body measurements were taken from Tanzanian chickens for their characterization (http://10.tbhy.allovero.fr/diagram-of-chicken-bone.html, 2/3/2020)

Linear measurements were obtained from chickens older than six months (mature chickens) and were performed as described by Geuye et al. [31]. In brief, the body length (BL), neck length (NL), chest girth (CG), shank length (SL) and shank girth (SG) were measured in centimetres (cm) for each chicken using a tailor’s measuring tape (Fig. 5) [32]. The body weights (BW) of the chickens were measured in grams (gm) using a 0.01 g sensitive electronic weighing scale. The linear measurements were performed as follows; BL was measured as the distance from tip of the beak through the dorsum of the chicken to the base of the tail, the NL from the base of the head to the shoulder at the clavicle, CG as the circumference of the chest in front of the thighs, SL as the distance from the hock joint to the metatarsal pad and the SC as the circumference of the middle part of the metatarsus.

### Population structure analysis

At 21 days of age, blood samples were collected from each chick by pricking the basilic vein. Approximately five drops of blood were dried on FTA cards (Whatman Biosciences, Brentford, UK) labelled with the chicken’s wing tag number and stored at room temperature. Section cuts (3 X 3 mm) using a scalpel blade were made in the cards for each chicken. The scalpel blade was decontaminated in between chickens via flaming. DNA was extracted and precipitated in sodium acetate ethanol using the phenol-chloroform method [33]. A total of 1399 birds were genotyped using a chicken 600 K SNP Panel at GeneSeek, USA, and quality control (QC) was performed using the Axiom™ Analysis Suite Software version 3.1 (Applied Biosystems, Thermo Fisher Scientific Inc., Calsbad, CA, USA) as explained by Walugembe et al. [34]. Briefly, *Gallus gallus* genome version 5 (Thermo Fisher Scientific Inc., Calsbad, CA, USA) chicken genome files were used for comparison during the genotyping of the FRLC. During quality control, SNP set with number of clusters ≥2, call rates ≥99% and minor allele frequency ≥ 0.05 were selected for downstream analyses. Other quality control metrics and imputation of missing genotypes are explained further in Walugembe et al. [34]. A total of 396,055 SNPs remained for further downstream analyses. Determination of the structure of the populations was performed using the admixture software [35] as explained in Walugembe et al. [34] where briefly, SNPs with closest ancestry were determined using varying values of k (sub-populations) ranging from 1 to 4 and the final k value (k = 2) was determined based on the lowest cross-validation error. The population structure was also determined using multi-dimensional scaling in plink [36] in two dimensions as shown by Walugembe et al. [34]. At the end of the studies, the chickens were humanely euthanized according to published guidelines [37] and the UC Davis IACUC (# 20831) protocol.

### Statistical analysis

The linear measurements were compared among the three chicken ecotypes using R - Statistical Software Program version 3.5.1 [38]. Analyses of variances (one – way ANOVA) of the least square means with associated standard errors (LSmeans±SE) of the measurements were used to assess for differences among the three chicken ecotypes. Differences were considered significant at *p* ≤ 0.05 using the Tukey honestly significant difference (Tukey HSD). The linear model to test the effects of the chicken ecotype and sex on the lengths of the measured body parts was as follows:

$$ {\mathrm{Y}}_{\mathrm{i}\mathrm{jk}}=\upmu +{\mathrm{G}}_{\mathrm{i}}+{\mathrm{A}}_{\mathrm{j}}+{\left(\mathrm{GA}\right)}_{\mathrm{i}\mathrm{jk}}+{\mathrm{e}}_{\mathrm{i}\mathrm{jk}} $$

where:

Y_ijk_ = trait response variable

μ = general population mean for trait response

G_i_ = effect of the sex on the trait of an ecotype

A_j_ = effect of the ecotype on the trait

(GA)ijk = effect of interaction between sex of chicken and its ecotype

e_ij_ = effect of random experimental errors on the trait response

## Data Availability

The datasets supporting the conclusions of this article are available in the USAID Data Development Library (DDL) repository, and can be accessed publicly at the https://www.usaid.gov/development-data-library request.

## Abbreviations

IACUC: Institutional Animal Care and Use Committee
FRLC: Free-range Local Chickens
ND: Newcastle disease
UCDavis: University of California Davis
USAID: United States Agency for International Development
DNA: Deoxyribonucleic Acid
SNP: single nucleotide polymorphism
MDS: multi-dimensional scaling
LSmeans: Least square means
Tukey HSD: Tukey honesty significant difference
ANOVA: Analyses of variance
QC: quality control
IBD: infectious bursal disease
SUA: Sokoine University of Agriculture
DAARS: Department of Animal, Aquaculture and Range Sciences
